# Supplementary material for: A translation proofreader of archaeal origin imparts multi-aldehyde stress tolerance to land plants
Source: eLife. 2024 Feb 19;12:RP92827. doi: 10.7554/eLife.92827 (PMC10942605; doi:10.7554/eLife.92827)
Supplement: Figure 4—source data 2. [file elife-92827-fig4-data2.pdf]

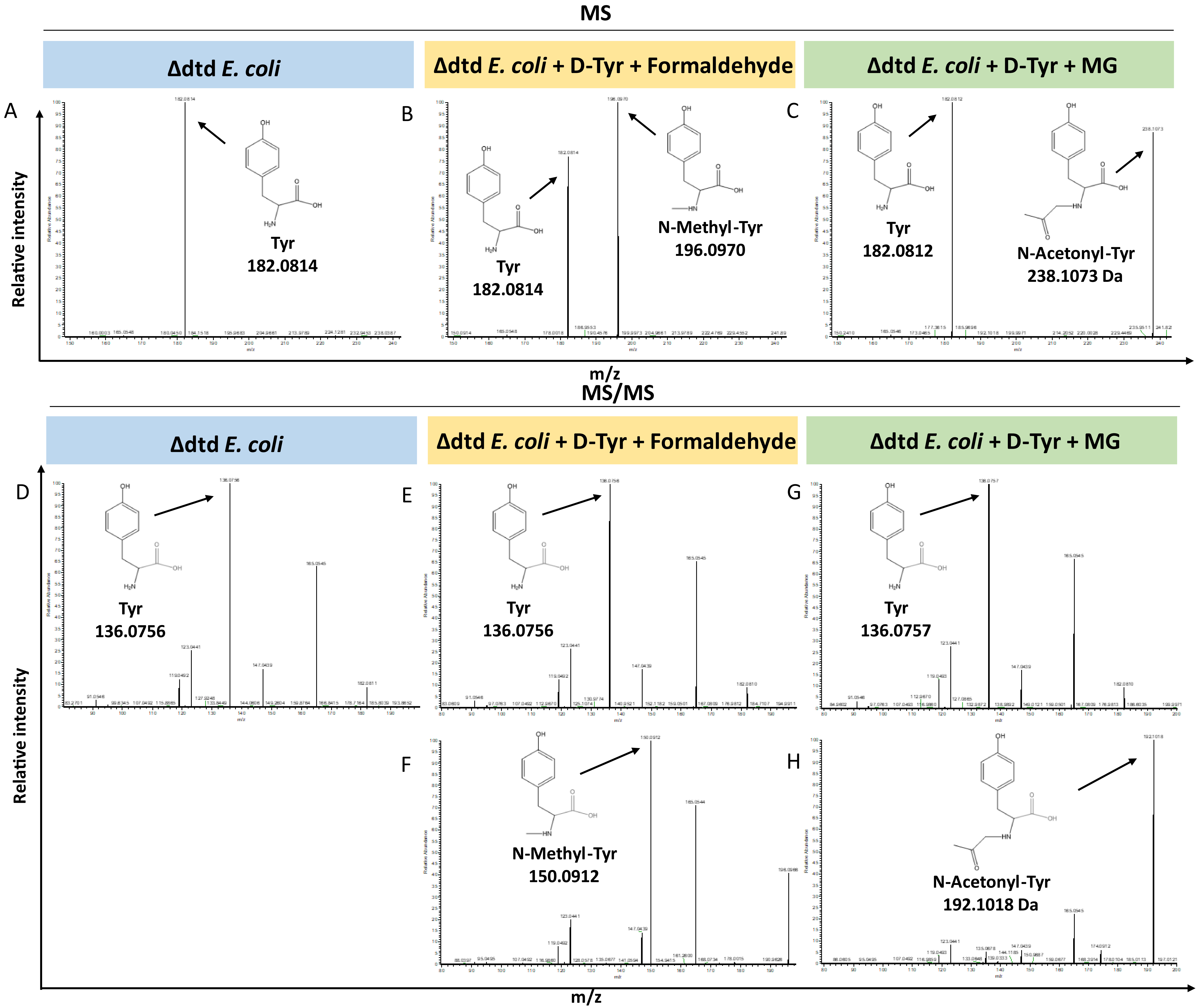
MS
Δdtd E. coli
Δdtd E. coli + D-Tyr + Formaldehyde
Δdtd E. coli + D-Tyr + MG
A
B
C
Relative intensity
Tyr
182.0814
Tyr
182.0814
N-Methyl-Tyr
196.0970
Tyr
182.0812
N-Acetonyl-Tyr
238.1073 Da
m/z
MS/MS
Δdtd E. coli
Δdtd E. coli + D-Tyr + Formaldehyde
Δdtd E. coli + D-Tyr + MG
D
E
G
F
H
Tyr
136.0756
Tyr
136.0756
Tyr
136.0757
N-Methyl-Tyr
150.0912
N-Acetonyl-Tyr
192.1018 Da
m/z
