# Supplementary figures and images for: A translation proofreader of archaeal origin imparts multi-aldehyde stress tolerance to land plants
Source: eLife. 2024 Feb 19;12:RP92827. doi: 10.7554/eLife.92827 (PMC10942605; doi:10.7554/eLife.92827)

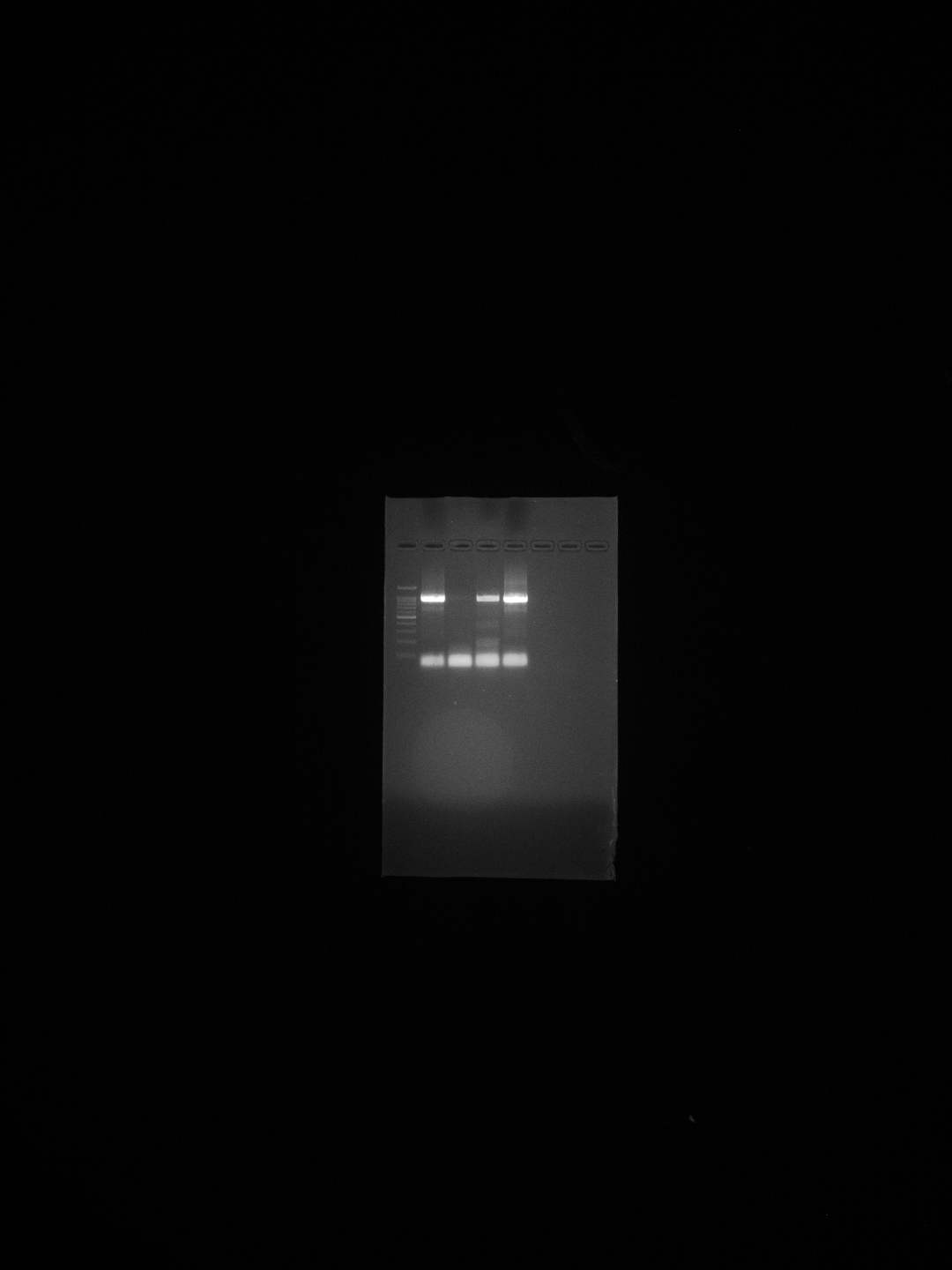

Supplement: Figure 4—source data 4. [file elife-92827-fig4-data4.zip › Source data used in Figure 4/Figure 4A_1.tiff]

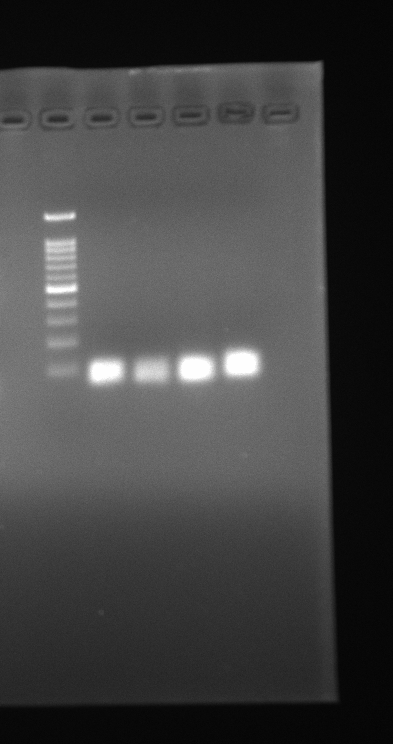

Supplement: Figure 4—source data 4. [file elife-92827-fig4-data4.zip › Source data used in Figure 4/Figure 4A_2.tiff]

Source data used in figure 4A

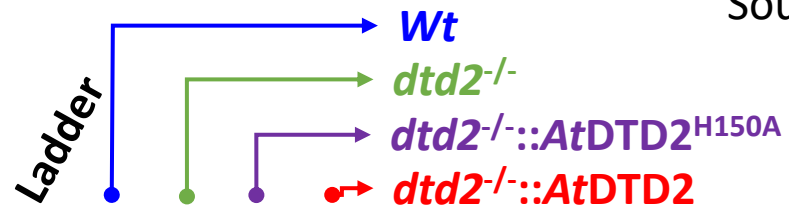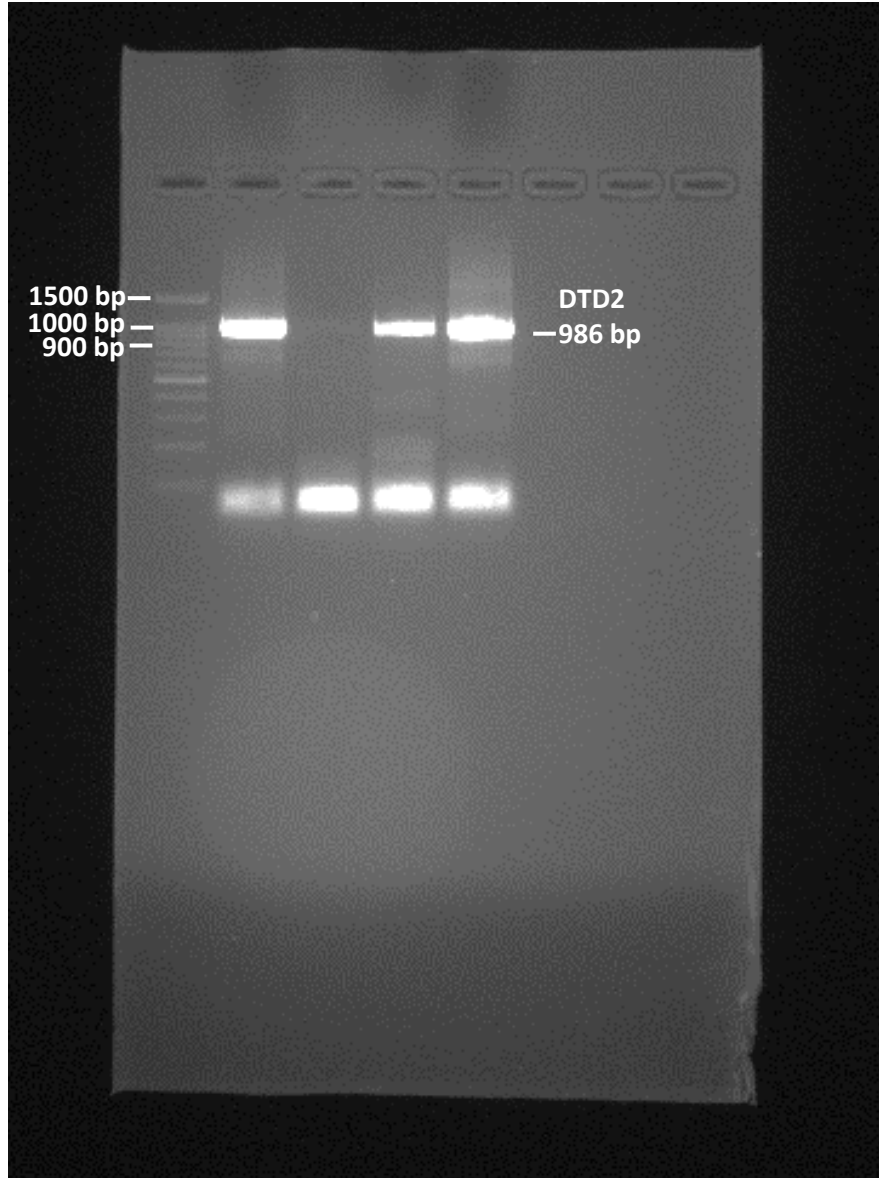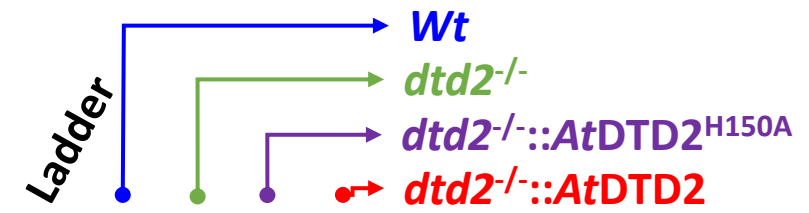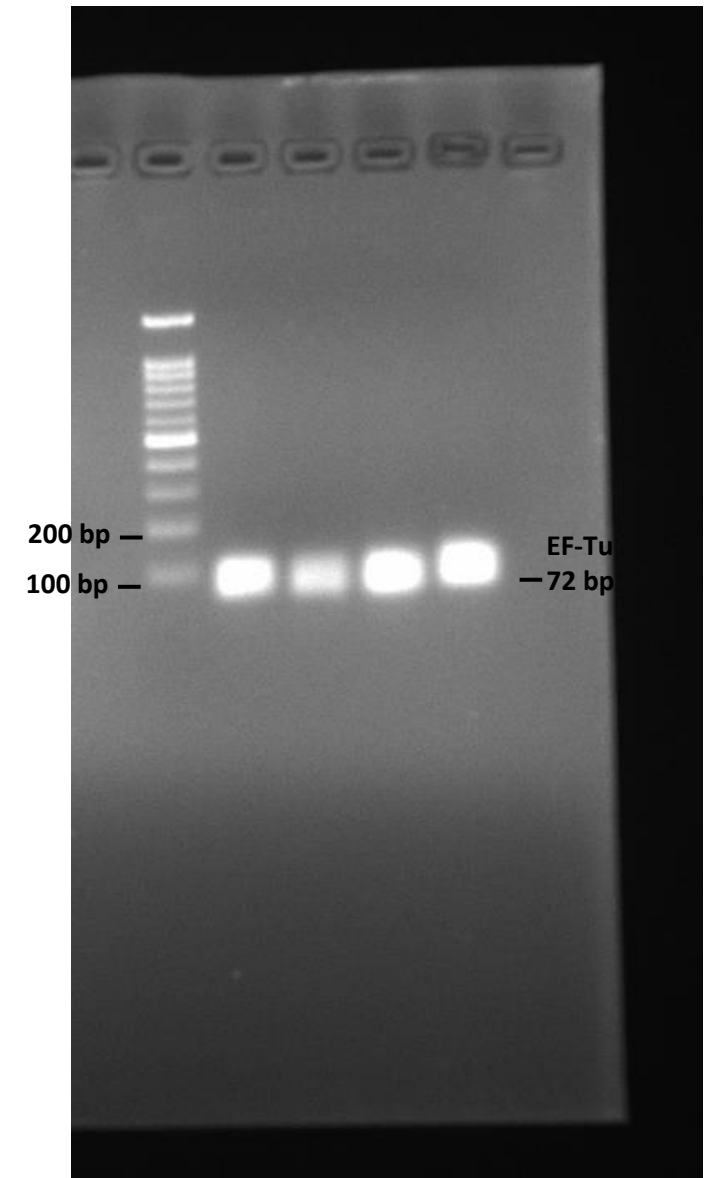

Supplement: Figure 4—source data 4. [file elife-92827-fig4-data4.zip › Source data used in Figure 4/Source data for Figure 4A.pdf]
